# 55-year-old Male with Bilateral Lower Extremity Weakness

**DOI:** 10.5811/cpcem.2017.8.35552

**Published:** 2017-11-03

**Authors:** Tejusve Rao, Anthony Roggio, Zachary D.W. Dezman, Laura J. Bontempo

**Affiliations:** * University of Maryland Medical Center, Baltimore, Maryland; † University of Maryland School of Medicine, Department of Emergency Medicine, Baltimore, Maryland

## CASE PRESENTATION

A 55-year-old male presented to a Level I trauma center via ambulance with a complaint of bilateral lower extremity weakness after falling. He stated he had slipped and fallen on his buttocks while showering. He discovered he was unable to stand, so he crawled to his bedroom and dialed 911. By the time the paramedics arrived to his home, he had no sensation or motor function below his knees bilaterally. A cervical collar was placed by the paramedics and the patient was transported to the hospital. Upon arrival, he continued to complain of pain to his buttocks. He denied any chest pain, shortness of breath, headache, syncope, abdominal pain, nausea, vomiting, or upper extremity weakness. He denied any past medical history or surgeries. He was not taking any medications and did not have any allergies. His family history was noncontributory. He denied smoking, alcohol, or any drug use.

Initial evaluation showed a well-developed, well-nourished male in no acute distress with a cervical collar in place. Triage vital signs were a temperature of 36.9° Celsius, heart rate of 77 beats per minute, respiratory rate of 23 breaths per minute, blood pressure of 139/95 millimeters mercury and pulse oximetry of 100% on room air. His body mass index was 23.79 kg/m^2^. His head was normocephalic and atraumatic. His pupils were equally reactive to light bilaterally with normal conjunctiva and sclera. His extraocular movements were intact. On cardiovascular exam, he had a regular rate and rhythm with normal heart sounds; specifically, no murmurs were auscultated. His upper extremity pulses were 2+ bilaterally, femoral pulses were 1+ bilaterally, and no dorsalis pedis or posterior tibial pulses were appreciated by palpation or with Doppler ultrasound. The patient was in no respiratory distress and his lungs were without wheezes, rhonchi or rales. His abdomen was soft and nontender with normal bowel sounds and no rebound or guarding. He had normal rectal tone but was not able to contract his anal sphincter on command.

Musculoskeletal exam had no cervical, thoracic or lumbar midline tenderness and no step-offs were palpated. He had normal range of motion throughout his bilateral upper extremities. Neurological exam revealed normal motor strength and reflexes throughout his bilateral upper extremities, but he was unable to move any portion of his bilateral lower extremities, including no ability to dorsiflex or plantarflex his feet. Patellar and ankle reflexes could not be elicited, and the plantar reflex was equivocal bilaterally. He had normal upper extremity sensation bilaterally but no sensory functions below his knees, including no sensation between his great and second toes. The patient did not have any nystagmus. He was alert and oriented to person, place and time and had no cranial nerve deficit. His skin was dry. His upper extremities were warm to touch and his lower extremities were cool to touch. A focused assessment with sonography in trauma (FAST) exam did not show any abnormalities. His laboratory values are shown in Tables 1–3. Based on the suspicion of the clinician, an additional test was done that confirmed the diagnosis.

## CASE DISCUSSION

The first thing I noted was that this patient was brought to the emergency department (ED) for bilateral lower extremity weakness of such severity that he had to *crawl* out of the bathroom. He reportedly has no sensation or ability to move below his knees. There are two important things to note right away: (1) this patient’s symptoms seemed to have happened suddenly; and (2) they happened *around* the time off the fall. The patient was routed by emergency medical services (EMS) to a Level I trauma center because they presumed a traumatic injury as the cause of his symptoms. However, I must not allow diagnostic inertia - in this case imposed by the EMS team’s assumption and the destination - to take hold. Keeping an open mind, the question arises: Which came first? Did he fall and then sustain neuromuscular weakness and numbness? Or did he develop sudden neuromuscular weakness and numbness, causing him to fall? My differential builds off of these two questions.

The patient’s symptoms suggest that I am well situated in a neurologic “box” of possible diagnoses. Listing causes of extremity weakness and numbness, I can begin with the central nervous system and move outward. Stroke and intracranial hemorrhage (traumatic or otherwise) come to mind, as well as more insidious mixed brain and spinal cord disorders such as multiple sclerosis or the central and peripheral nerve effects of amyotrophic lateral sclerosis. In this list as well are brain and spinal cord tumors, complex migraines with neurologic deficits, seizures with Todd’s paralysis, and infectious possibilities such as meningitis and encephalitis.

Further down the central nervous system, injuries of the spinal cord prevail. In this list are traumatic injuries such as traumatic disk herniation with sciatica, as well as spinal fractures and the spectrum of cord injuries such as Brown-Séquard’s hemisection, anterior and posterior traumatic cord injuries, cord contusion and spinal cord injury without radiographic abnormality. Added to this list are transverse myelitis, spontaneous or traumatic hemorrhage compressing the spinal cord, various causes of loss of circulation to the spinal cord such as embolism or vascular rupture, the dreaded epidural abscess, and the feared cauda equina.

In the peripheral nervous system I consider distal nerve disorders such as Guillain-Barré, neuromuscular endplate disorders, and myasthenic crisis, to name a few.

Furthermore, I cannot forget the toxic, metabolic, and endocrine causes of neurologic dysfunction as well. Hypokalemic periodic paralysis, severe hypo/hypernatremia, hypo/hypercalcemia, hypophosphatemia, hypoglycemia, hyperglycemic nonketotic syndrome, botulinum toxin, and ciguatera poisoning are all of concern.

With my differential in hand, I tackle the remainder of the history – which is significantly insignificant. While this could mean he hasn’t seen a doctor in the last 55 years of his life, I will take it at face value. Unless it is a new diagnosis, this lowers suspicion for more chronic disorders, which one would imagine should have at least hints of symptoms before this point.

In the patient’s review of systems there is much to highlight. He had no fevers or recent illnesses or cold symptoms, lowering infectious causes such as an epidural abscess on my differential and decreasing my worry for Guillain-Barré (though the timing of illness to onset of symptoms may be prolonged). Suspicion for meningitis and encephalitis is also lessened with this information.

He notes lower back and buttock pain, but no headache, seizure, syncope, or lightheadedness. Also his weakness and numbness is bilateral. This particular set of information shuffles diagnostic likelihoods in my differential considerably. Lower back and buttock pain may be expected after a fall, and potentially escalates traumatic injury on my differential diagnosis list. In an alert patient without any headache, the patient is unlikely to have a complex migraine with neurologic deficit or intracranial bleeding. Todd’s paralysis is essentially removed from my thought process without a seizure. Suspicion for meningitis and encephalitis is similarly lowered without headache. The possibility of thromboembolic stroke is also lessened as few strokes can cause bilateral symptoms, and those that do would be presumably large-area strokes with multiple vessel occlusions likely affecting more than just the lower extremities.

I have whittled my differential diagnoses considerably with history alone. Some questions still remain unanswered, however. Exactly what areas of the body are affected by “numbness” and “weakness?” Are they equal bilaterally or is one side worse than the other? Does the deficit follow a dermatomal distribution? Are there signs of spinal cord injury? Are the patient’s symptoms improving? I remind myself of the increased reflex spasticity in upper motor neuron lesions compared to lower motor neuron lesions and hope that I can find a reflex hammer (or suitable approximation) nearby. I move on to the physical exam and specifically the neurologic examination to help answer these questions.

On initial review, aside from mild blood pressure elevation and respiratory rate elevation, vitals are essentially normal. I focus intently on the patient’s trauma and neurologic examinations. Of particular note, the patient has *no* spinal tenderness on exam and no palpable step offs/injuries. This goes against traumatic spinal cord injury but does not completely remove it from my thought process. Traumatic fracture or subluxation and related entities such as cord transection move down slightly in my differential.

In terms of motor function, the patient has normal tone on rectal exam but is unable to squeeze on command, and his lower extremities are completely unable to move distal to the knees. He is also reportedly completely devoid of sensation in the same area. This is extremely important information, because while neurologic deficits below the knees could be due to a range of central or peripheral issues, the fact that his voluntary rectal muscle control (controlled by the sacral nerves) is affected as well allows me to conclude that his deficits are dermatomal at the lumbar 4–5 (L4–5) vertebral level and below. Could there be such a significant spinal cord injury without palpable abnormality? Perhaps in the case of contusion and hemorrhage. I again seem to be pointed to traumatic injury of the cord and move this set of diagnoses higher on the differential list. That is, until the peripheral cardiovascular exam…

The patient has normal upper extremity pulses but decreased femoral and absent dorsalis pedis and posterior tibial pulses. The patient has no diagnosed medical problems and no previous report of arterial disease (cardiac or peripheral). Why then does he have absent distal lower extremity pulses in the same areas he has acute neurologic complaints?

Looking at his lab work, a lactic acid of 6.6 supports that these findings are likely related to an *acute injury* resulting in ischemia (while his otherwise nonspecific labs help remove a significant portion of the toxic and metabolic components of my differential).

Immediately, alarm bells ring in my mind as an acute loss of pulses sends shockwaves through the differential, removing or significantly deprioritizing a considerable fraction of potential diagnoses. Disease processes that don’t include vascular abnormalities are completely removed from my mind in this instance, eliminating cauda equina, Guillan-Barré, transverse myelitis, brain tumors, distal neuron or endplate disorders, and the like. In breaking down the possible diagnoses for acute loss of pulses, I remember the four essential vascular causes by introducing the mnemonic “RODE.” I must test the patient’s symptoms and physical exam findings against these possibilities:

- Rupture
- Occlusion (includes thromboembolism)
- Dissection
- External compression (includes compartment syndrome)

### Rupture

It is possible a ruptured abdominal aortic aneurysm (AAA) could present with loss of pulses and ischemia. However, the history doesn’t fit the classic story of AAA rupture. The patient has no abdominal pain that is typically associated with the disease and no history of hypertension or connective tissue disease, which are typically needed for an aneurysm to develop. In a significant rupture causing vascular and neurologic deficits, I would expect the patient to show signs of shock or sudden blood loss on exam, such as hypotension, pallor, and diaphoresis, of which there is no mention.

Also, while this diagnosis would explain his diminished femoral pulses and absent pedal pulses, it would not necessarily explain the dermatomal distribution of his neurologic deficits - if the patient has femoral pulses, we would expect the blood flow to the spinal arteries (which have a more proximal takeoff on the aorta) to continue to be adequate. An alternative and perhaps more reasonable explanation would be that if the patient did fracture and sublux his lumbosacral spine in the fall, he could have completely torn the radiculolumbosacral arteries or posterior spinal arteries feeding the spinal cord. This would account for the dermatomal distribution of his symptoms but it *would not* explain why the pulses were diminished in the lower extremities. Furthermore, there was no significant step off palpated in the spine exam to corroborate this line of thinking.

### Occlusion and Dissection

In considering dissection and thromboembolic disease, I have to take anatomy into account. The legs are individually supplied by the femoral arteries (rising from the iliac arteries), which split into the superficial and common femoral arteries and then divide further as you get more distal. Multiple distal emboli as the cause of the patient’s symptoms are a possibility. However, the patient has intact but diminished femoral pulses, signifying the vascular abnormality begins more centrally. A large complete central thrombus or dissection is possible, but this should make femoral pulses disappear and you would expect more signs of severe ischemia to the lower extremities such as mottling, cyanosis or pallor, and more lower extremity pain as well. If there is an occlusion or dissection, it is likely only partial.

### External compression

Could the patient have bilateral lower extremity compartment syndrome? Aside from no reported lower extremity trauma or crush injury and no swelling on exam, I consider the “5 P’s” of this diagnosis:

- Pain
- Pallor
- Poikilothermia
- Paresthesias
- Pulselessness

The patient has only three of the 5 P’s– poikilothermia, paresthesias, and pulselessness. He is not complaining of significant pain to his lower extremities and there is no reported mottling or pallor of the skin. While you don’t need all five signs to make a diagnosis, pulselessness is typically a late finding occurring after the others. This diagnosis is unlikely.

So using the mnemonic, I have tackled each vascular abnormality on its own and come up with little to explain diminished blood flow to the lower extremities leading to his neurologic symptoms. Remember the patient’s neurologic deficits appear to have a *dermatomal distribution* localized to the L4–5 level and below, meaning there must be spinal cord involvement.

How then can I marry the vascular finding of diminished pulses and neurologic findings of an insult at the L4–5 spinal cord: By narrowing my gaze directly to the site where the two unite – the vascular supply to the spinal cord.

With all other options on my differential accounted for, the combination of vascular symptoms and dermatomal distribution of neurologic abnormalities leads me to the only conclusion that will explain all symptoms – the patient has a loss of blood flow to the spinal cord at the L4–5 level. So which of the four “RODE” possibilities for loss of blood flow would account for that?

Based on the patient’s complaint of low back pain without significant traumatic injury, and the finding of diminished pulses distally, this spinal cord infarction is most likely from an aortic dissection occluding the spinal arteries and partially extending into the iliacs. When considering dissection, point-of-care ultrasound (POCUS) can help diagnose this condition; however, it is limited to anatomically accessible portions of the great vessels, is provider and experience dependent, and is more prone to error or missed diagnosis. Given this low sensitivity, a positive POCUS is useful to mobilize a surgical and/or vascular team quickly but may not adequately demonstrate the extent of disease; and a negative ultrasound *cannot* rule out the diagnosis. The test of choice, therefore, is a computed tomography angiogram (CTA) of the chest, abdomen and pelvis.

## CASE OUTCOME

A CTA of the patient’s chest, abdomen, and pelvis revealed a large Type A aortic dissection with hemopericardium. This patient’s dissection extended into the great vessels of the neck and the descending aorta. The dissection extended into the right renal, celiac, and superior mesenteric arteries with thrombosis of the lower abdominal aorta and left iliac artery. The thrombosis likely caused decreased flow to the spinal arteries and was the source of the patient’s lower extremity weakness. Cardiothoracic surgery and vascular surgery were immediately notified, while infusions of esmolol and nicardipine were started to slow the patient’s heart rate and lower his blood pressure.

The patient underwent emergency surgery for the placement of a thoracic endovascular aortic graft into the descending aorta and an ascending interposition graft. His aortic valve was re-suspended and the patient was given a left femoral to right femoral bypass with right iliac angioplasty and stenting (Image). He also required bilateral lower extremity fasciotomies. The patient did well during the immediate post-operative period and had closure of his fasciotomies a few days later. He was treated with beta blockers and amiodarone for blood pressure and rhythm control. A month after his initial presentation, he was discharged to home with regular home health visits.

## RESIDENT DISCUSSION

Aortic dissection is a life-threatening emergency with high rates of morbidity and mortality. Since this illness is rapidly fatal, the incidence is difficult to obtain. However, some studies have noted the incidence to be about two to 3.5 cases per 100,000 people.^1^–^3^ The mean age of a patient with acute aortic dissection is 63.1 years and about two thirds of patients are male.^4^,^5^ Women with dissections tend to be older and have higher mortality rates than men.^6^ The most common pathophysiologic process that occurs is an intimal tear, which creates a false lumen where blood can propagate in an anterograde or retrograde fashion. Intimal tears can also arise from atherosclerotic ulcers or a traumatic injury.^4^,^7^

The patients usually have a history of hypertension among other risk factors, which include prior cardiac surgery, atherosclerosis, connective tissue disorders such as Marfan syndrome or Ehlers-Danlos syndrome, family history, and known aortic aneurysm.^4^,^8^–^10^ Although the classic presentation has been described as chest pain that is tearing or ripping in nature, the abrupt onset of severe, “worst-ever” pain is the most common historical finding (90%).^4^,^8^,^9^ Presentations can vary because the false lumen can occlude any of the branching arteries along the aorta. Patients can present with chest pain radiating to the back or abdomen, but they can also have chest pain radiating below the diaphragm, chest pain with neurologic deficits, or chest pain associated with syncope and pulse deficits.^9^,^11^ There are reports of acute aortic regurgitation, myocardial ischemia or infarction, heart failure and shock, pericardial effusion and tamponade, paraplegia secondary to spinal cord malperfusion, and mesenteric ischemia.

Initial testing such as chest radiography (CXR) or electrocardiogram can be very nonspecific. The classic presentation of mediastinal widening or abnormal aortic contour were absent in 37.4% of patients; thus, a CXR is not sensitive enough to definitively exclude a dissection.^4^ If a patient is determined to be high risk, a negative CXR should not delay you from obtaining definitive aortic imaging. Electrocardiography can be normal or show nonspecific changes in 31.3% of patients.^4^,^6^,^11^ Other diagnostic modalities such as echocardiography or magnetic resonance imaging/magnetic resonance angiography (MRI/MRA) can detect an aortic dissection, but CTA is the diagnostic test of choice. The sensitivities and specificities of all three modalities approach 100%.^1^ The advantages of a CTA include the almost universal availability, short acquisition time, and high accuracy. A potential pitfall is to focus imaging on one body region. Because a dissection can occur at any point along the aorta, a complete evaluation has to include imaging of the chest, abdomen, and pelvis.

The classifications that are used to characterize the type of aortic dissection are the Stanford, DeBakey, and Svensson.^12^ In the Stanford classification, which is more commonly used, Type B dissections involve the descending aorta whereas Type A involve the ascending and possibly the descending aorta. Irrespective of the anatomic location of the dissection, the American Heart Association (AHA) recommends urgent surgical consultation.^1^ Both Type A and Type B aortic dissections require aggressive medical management including blood pressure reduction with beta blockers, or non-dihydropyridine calcium channel blockers intravenously to reduce the shear forces and aortic wall stress.^12^ Most patients with Type A aortic dissections are managed surgically^12^ and approximately 80% of Type B dissections are treated medically.^4^ The mortality rates continue to be high despite advances in imaging and medical therapy.

The AHA and the American College of Cardiology (ACC) in 2010 proposed the Aortic Dissection Detection Risk Score that risk stratified patients based on low, intermediate, and high probability of aortic dissection.^1^ Subsequent studies have shown that 4.3% of patients with aortic dissection were classified as low risk using this risk-scoring system.^13^ The American College of Emergency Physicians’ guidelines recommend against using these clinical decision rules, and suggest that the decision to pursue a further workup should be at the discretion of the treating physician (Evidence level C).^14^ There have been studies to evaluate the use of D-dimer for screening individuals if clinical suspicion exists for aortic dissection; however, the AHA and the ACC do not recommend routine serum D-dimer screening for patients being evaluated for aortic dissection.^1^

## FINAL DIAGNOSIS

Aortic Dissection

## KEY TEACHING POINTS

1. Aortic dissection is a life-threatening medical emergency with a variety of presentations. Abrupt onset of severe chest pain is the most common presenting symptom.
2. Chest pain associated with syncope, neurologic deficits or any pulse deficits should raise suspicion for aortic dissection.
3. Imaging modalities include CTA, echocardiography, and MRI/MRA. CTA is fast, accurate, and widely available. It is the diagnostic test of choice.
4. Once an aortic dissection is confirmed, prompt surgical consultation and aggressive medical management is required.

## Figures and Tables

**Image:**
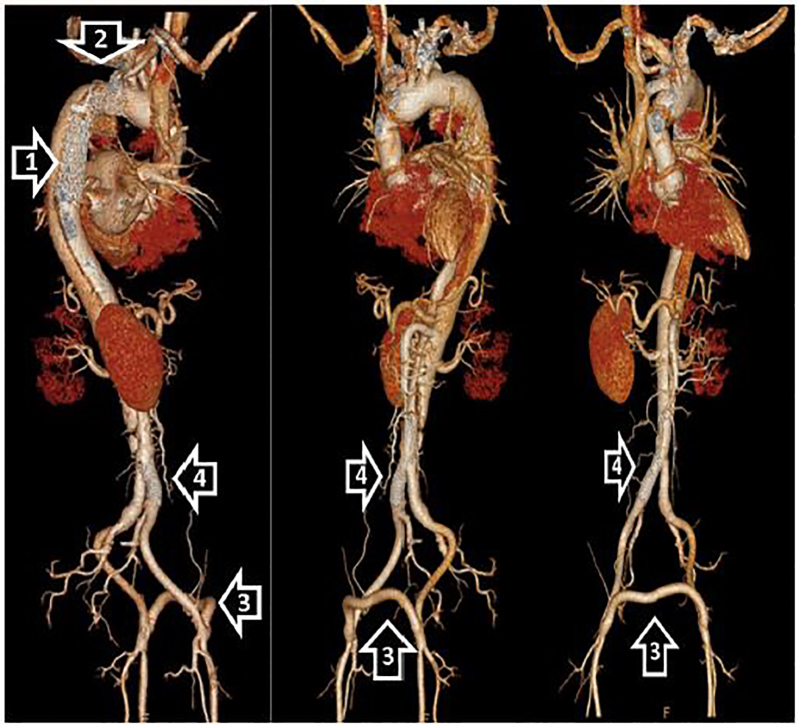
Multiple three-dimensional reconstruction views of computer tomography angiogram of the aorta demonstrating the thoracic endovascular aortic graft (1) into the descending aorta, and an ascending interposition graft (2). Including the left femoral to right femoral bypass graft (3), and right iliac stent (4).

**Table 1:** Hematology, chemistry and cardiac studies in patient with bilateral lower extremity weakness.

White blood cell count	19.6 K/mcL
Hemoglobin	10.8 g/dL
Sodium	142 mmol/L
Potassium	3.8 mmol/L
Chloride	109 mmol/L
Aspartate aminotransferase	31 units/L
Alanine aminotransferase	16 units/L
Alkaline phosphatase	59 units/L
Anion gap	17*
Troponin	<0.02 ng/mL
Hematocrit	32.3%
Platelets	190 K/mcL
Bicarbonate	16 mmol/L
Blood urea nitrogen	18 mg/dL
Creatinine	1.06 mg/dL
Glucose	158 mg/dL
Magnesium	1.8 mEq/L
Phosphorus	3.4 mg/dL
Lactate	6.6 mmol/L
CK MB	0.6 ng/mL

*CK,* creatine kinase; MB, muscle and brain.

* Normal range: 4–16.

**Table 2:** Urinalysis and toxicology screen.

pH	6.0
Color	Straw
Blood	Trace
Glucose	Trace
Acetaminophen	< 10.0 mcg/mL
Salicylate	< 1.0 mg/dL
Ethanol	< 10 mg/dL
Benzodiazepine	Negative
Barbiturates	Negative
Tricyclic	Negative
Red blood cells	11–25 count/uL
White blood cells	0–2 count/uL
Bacteria	Trace
Squamous epithelial	Negative
Amphetamine	Negative
Cannabinoid	Negative
Cocaine	Negative
Methadone	Negative
Phencyclidine	Negative
Opiates	Positive

**Table 3:** Coagulation studies and thromboelastography.

Prothrombin time	14.7 sec
International normalized ratio	1.1
TEG clotting time	3.1 minutes
TEG K time	1.1 minutes
TEG fibrinogen activity: (angle)	73.7 degrees
Activated partial thromboplastin time	28 sec
TEG coagulation index	3.5
TEG LYSE30	0.0%
TEG platelet aggregation: (MA)	66.3mm

*TEG,* thromboelastography.
